# Macrophage-derived interleukin-6 is necessary and sufficient for choroidal angiogenesis

**DOI:** 10.1038/s41598-021-97522-x

**Published:** 2021-09-10

**Authors:** Steven Droho, Carla M. Cuda, Harris Perlman, Jeremy A. Lavine

**Affiliations:** 1 Department of Ophthalmology, Feinberg School of Medicine, Northwestern University, Chicago, IL USA; 2 Department of Medicine, Division of Rheumatology, Feinberg School of Medicine, Northwestern University, Chicago, IL USA

**Keywords:** Monocytes and macrophages, Macular degeneration

## Abstract

Neovascular age-related macular degeneration (nAMD) commonly causes vision loss from aberrant angiogenesis, termed choroidal neovascularization (CNV). Interleukin-6 (IL6) is a pro-inflammatory and pro-angiogenic cytokine that is correlated with AMD progression and nAMD activity. We hypothesize that anti-IL6 therapy is a potential nAMD therapeutic. We found that IL6 levels were increased after laser injury and expressed by macrophages. *Il6*-deficiency decreased laser-induced CNV area and exogenous IL6 addition increased choroidal sprouting angiogenesis. *Il6*-null mice demonstrated equally increased macrophage numbers as wildtype mice. At steady state, IL6R expression was detected on peripheral blood and ocular monocytes. After laser injury, the number of IL6R^+^Ly6C^+^ monocytes in blood and IL6R^+^ macrophages in the eye were increased. In human choroid, macrophages expressed *IL6*, *IL6R*, and *IL6ST*. Furthermore, *IL6R*^+^ macrophages displayed a transcriptional profile consistent with STAT3 (signal transducer and activator of transcription 3) activation and angiogenesis. Our data show that IL6 is both necessary and sufficient for choroidal angiogenesis. Macrophage-derived IL6 may stimulate choroidal angiogenesis via classical activation of IL6R^+^ macrophages, which then stimulate angiogenesis. Targeting IL6 or the IL6R could be an effective adjunctive therapy for treatment-resistant nAMD patients.

## Introduction

Age-related macular degeneration (AMD) is the most common cause of vision loss in the developed world. Neovascular AMD (nAMD) occurs when angiogenesis from the choroidal vasculature invades through Bruch’s membrane into the sub-retinal pigment epithelium (RPE) or sub-retinal space, a destructive process termed choroidal neovascularization (CNV). Current nAMD treatment blocks angiogenesis by inhibiting vascular endothelial growth factor (VEGF). Intravitreal anti-VEGF injections improve vision by 5–10 letters^1^; however, frequent injections are expensive, include the risk of endophthalmitis, and 15% of patients lose vision despite monthly therapy^2^. Therefore, an unmet need exists for alternative therapies.

Our group, and several other labs, have demonstrated that beta-adrenergic receptor blockade inhibits experimental CNV by 50–80%^3–8^. These findings led to a randomized, placebo controlled clinical trial. Patients with treatment-resistant nAMD received continued monthly anti-VEGF injections plus either placebo or topical dorzolamide-timolol (carbonic anhydrase inhibitor plus beta-adrenergic receptor blocker). Adjuvant topical dorzolamide-timolol significantly reduced persistent retinal fluid compared to placebo^9^, an important finding to help reduce anti-VEGF injection burden. Despite these results, the mechanism by which beta-adrenergic receptor blockade inhibits CNV remains unknown.

Interleukin-6 (IL6) is a pro-inflammatory cytokine produced by macrophages. Classical monocyte-derived macrophages are necessary for propranolol-driven CNV blockade^6^, and propranolol decreases IL6 levels during experimental CNV^3,4^. These data implicate macrophage-derived IL6 as a key driver of experimental CNV. In support, IL6 stimulates angiogenesis via both VEGF dependent and independent pathways^10^. Based upon these results, we investigated the role of IL6 during experimental CNV.

We found that IL6 expression was increased in macrophages at the CNV lesion during the height of macrophage infiltration. IL6 knockout mice demonstrated reduced CNV area compared to wildtype mice, and were no longer susceptible to propranolol-driven CNV inhibition. Additionally, IL6 was sufficient to stimulate choroidal angiogenesis. Furthermore, the IL6 receptor (IL6R) was expressed on peripheral blood and ocular monocytes at steady state. After laser injury, the number of IL6R^+^Ly6C^+^ monocytes increased in peripheral blood, and IL6R^+^ macrophages were detected in the eye. Similar to mice, human choroidal macrophages expressed IL6 and the IL6R. IL6R^+^ human macrophages showed a transcriptional profile consistent with signal transducer and activator of transcription 3 (STAT3) activation and angiogenesis. These data suggest that macrophage-produced IL6 stimulates choroidal angiogenesis by classically activating IL6R^+^ macrophages.

## Results

**Figure 1:**
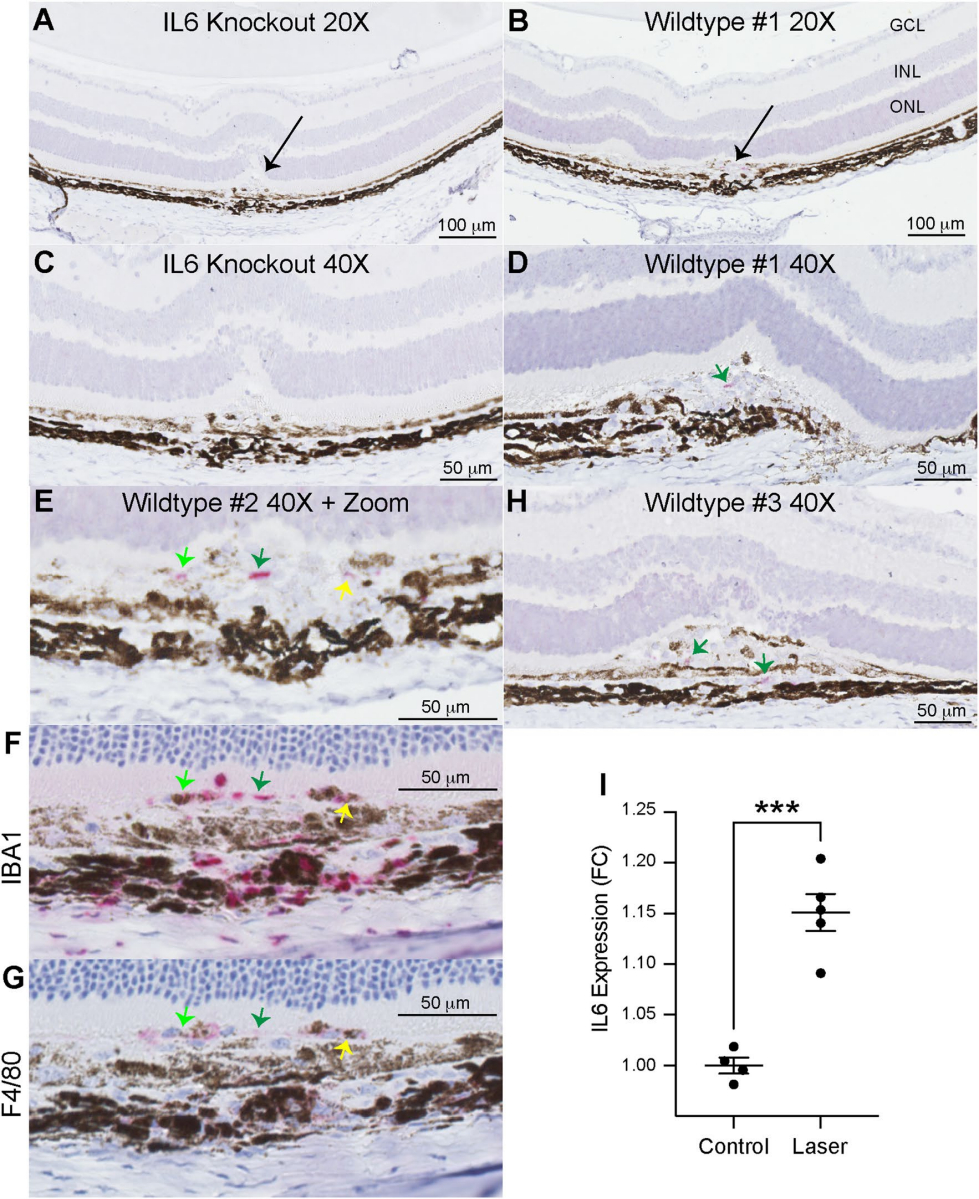
IL6 expression is increased by laser and expressed at the laser injury site. 20X magnification of paraffin-embedded sections stained for hematoxylin and IL6 (pink, RNAscope) from *Il6*^−/−^ (**A**) and wildtype (**B**) mice. Arrows indicate laser lesion. 40X magnification of sections stained for hematoxylin and IL6 (pink, RNAscope) from *Il6*^−/−^ (**C**) and wildtype (**D**, **H**) mice. Green arrows indicate IL6 + cells. 40$\times$ magnification with digital zoom of IL6 (**E**), IBA1 (**F**), and F4/80 (**G**) in serial sections from a single lesion. Light green, dark green, and yellow arrows indicate IL6 + cells co-staining for IBA1 and F4/80 in serial sections. ELISA measurements (**I**) of IL6 expression demonstrate a 1.15-fold (*** = p < 0.001, N = 4–5 per group) increase after laser injury.

Since IL6 is a secreted molecule, we used RNAscope to identify IL6-producing cells. In 10–12 week-old female wildtype and *Il6*^−/−^ mice, we performed laser injury and harvested eyes on Day 3, the peak of macrophage recruitment^11^. We found no IL6 expression in *Il6*^−/−^ mice, confirming the validity of the RNAscope probe (Fig. 1a,c). In wildtype mice, IL6 expression was only detectable at the laser injury site (black arrow, Fig. 1b). In three independent wildtype mice, IL6^+^ cells (pink stain, green and yellow arrows) were found in the inflammatory lesion (Fig. 1d,e,h). Next, we performed immunohistochemical staining of serial sections (4 $\upmu$m sections, 2–4 sections apart) for IBA1 and F4/80 from Wildtype #2 (Fig. 1e) to identify if IL6^+^ cells were macrophages. We found both IBA1^+^ and F4/80^+^ staining at near identical locations to IL6^+^ cells (Fig. 1e–g, colored arrows). In order to quantitatively confirm these results, we performed IL6 ELISA on posterior eye cups (retina and choroid-RPE-sclera complex). IL6 levels were increased 1.15-fold (p < 0.001) on Day 3 after laser injury (Fig. 1i). These data demonstrate that macrophages produce IL6 at the site of laser injury.

**Figure 2:**
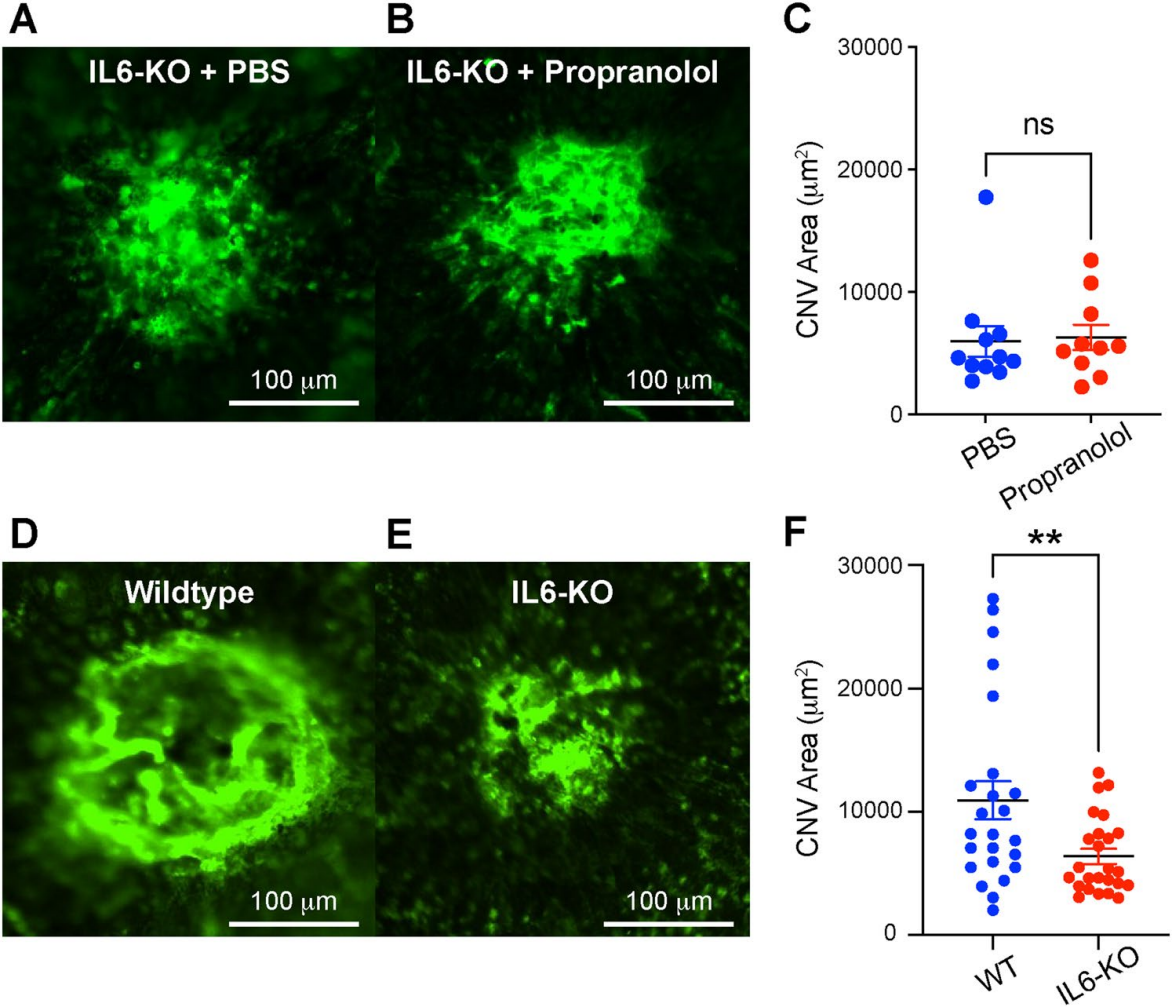
IL6 is necessary for CNV. Representative immunofluorescence staining of CNV lesions in PBS (**A**) and propranolol (**B**) treated *Il6*^−/−^ mice. Propranolol had no effect upon CNV area in IL6-null mice (ns = not significant, N = 10–11 per group, C). Representative immunofluorescence staining of CNV lesions in wildtype (D) and *Il6*^−/−^ (E) mice. IL6-deficiency resulted in a 42% decrease in CNV area (** = p < 0.01, N = 24–25 per group, F).

IL6 is increased after laser injury (Fig. 1), and reduced by propranolol^3,4^. We next investigated whether IL6 is necessary for propranolol-driven CNV blockade. We subjected 10–12 week old female *Il6*^−/−^ mice to laser injury. Female mice were used because we previously demonstrated that male mice do not demonstrate propranolol-induced CNV blockade^6^. Mice received daily intraperitoneal vehicle (PBS) or propranolol (20 mg/kg) injections for 14 days. In the context of IL6-deficiency, propranolol had no effect upon CNV area (Fig. 2a–c). Next, we investigated if IL6 deficiency affects CNV compared to wildtype mice. We subjected male and female 10–12 week old wildtype and *Il6*^−/−^ mice to laser injury and quantified CNV area on Day 14. IL6-deficient mice displayed a 42% reduction in CNV area (p < 0.01, Fig. 2d–f) with no sex-dependent effects (Fig S1). These findings show that IL6 is necessary for both propranolol-driven CNV blockade and CNV pathogenesis.

**Figure 3:**
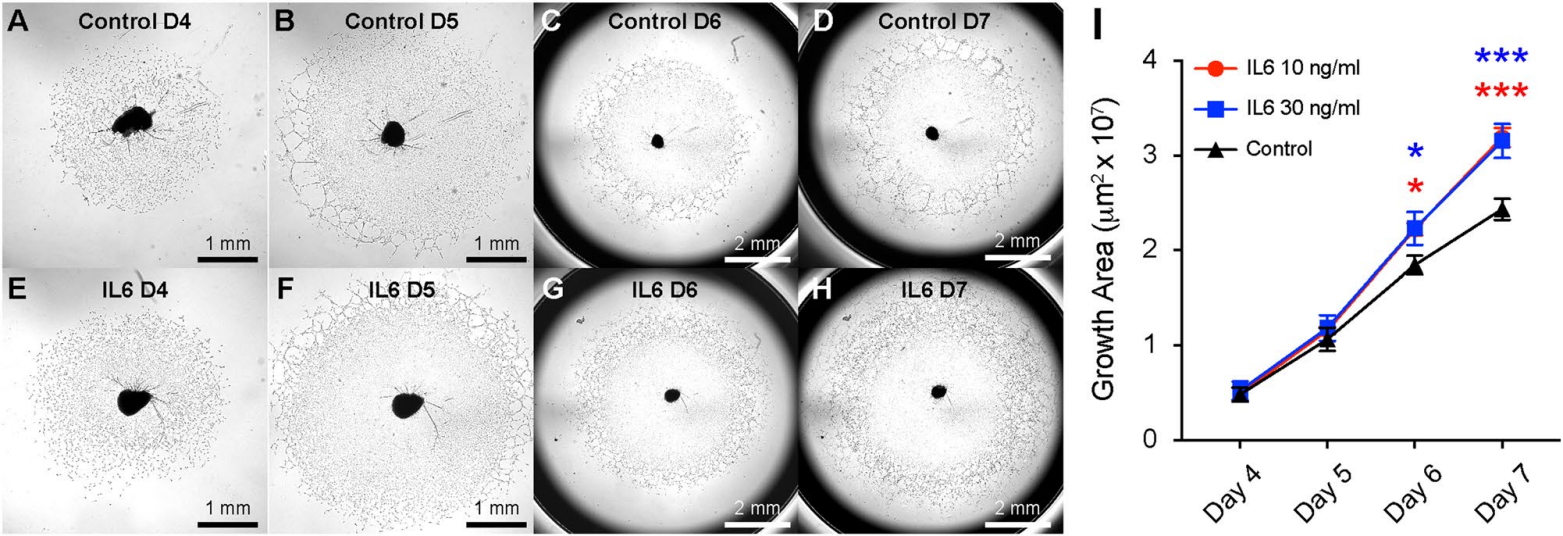
IL6 is sufficient for choroidal angiogenesis. Representative phase contrast brightfield microscopy of choroidal sprouts from control (**A**–**D**) and IL6 (**E**–**H**) treatment on Days 4 – 7. Exogenous IL6 treatment (**I**) increased choroidal angiogenesis area by 1.2 fold (* = p < 0.05, N = 5 per group) on Day 6 (D6) and 1.3 fold (*** = p < 0.001) on Day 7 (D7).

IL6 is capable of directly stimulating angiogenesis outside the eye^10^. To test this effect in the choroid, we performed ex vivo choroidal sprouting assays in the presence of vehicle (Fig. 3a–d) or exogenous IL6 (Fig. 3e–h). Direct addition of IL6 at 10 and 30 ng/ml increased choroidal angiogenesis area by 1.2-fold (p < 0.05 for both) on Day 6, and 1.3-fold (p < 0.001 for both) on Day 7 (Fig. 3i). These data demonstrate that IL6 is sufficient to stimulate choroidal angiogenesis.

**Figure 4:**
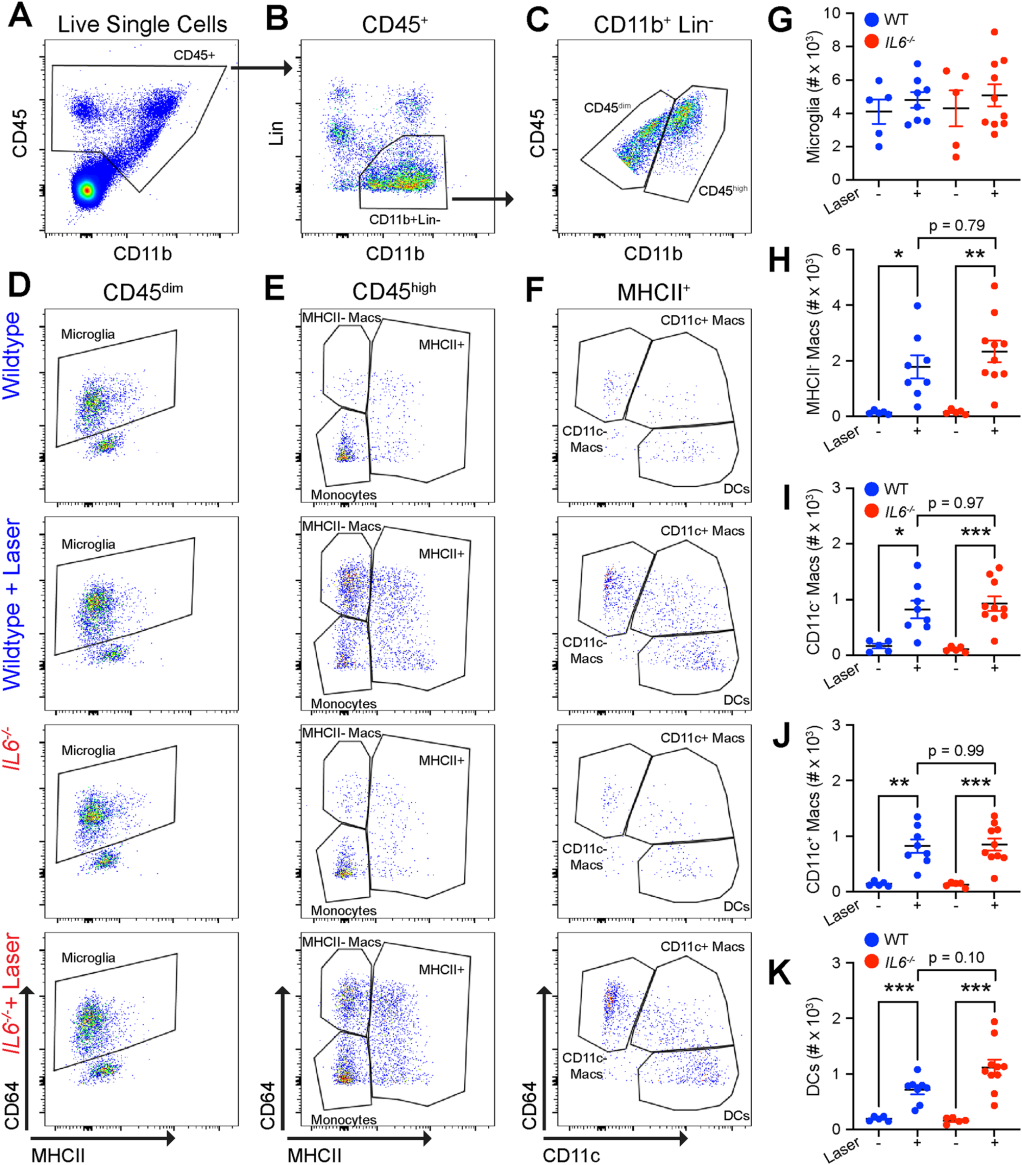
Wildtype and *Il6*^−/−^ mice demonstrate similarly increased macrophage numbers. (**A**) CD45^+^ cells were identified from live and singlet cells. (**B**) Lineage (Lin) gate was used to exclude neutrophils (Ly6G), eosinophils (SiglecF), NK cells (NK1.1), T cells (CD4, CD8), and B cells (B220), and gate forward CD11b^+^Lin^−^ mononuclear phagocytes. (**C**) Quantitative CD45 expression was used to separate CD45^dim^ from CD45^high^ cells. (**D**) Microglia were defined as CD45^dim^CD64^+^MHCII^low^ from wildtype and *Il6*^−/−^ mice both with and without laser. (**E**): MHCII^−^ macrophages (macs) were delineated as CD45^high^CD64^+^MHCII^−^, and CD45^high^MHCII^+^ cells were gated forward. (**F**): CD11c^−^ and CD11c^+^ macrophages were identified by CD64^+^MHCII^+^CD11c^−^ and CD64^+^MHCII^+^CD11c^+^, respectively. Dendritic cells (DC) were designated as CD64^−^MHCII^+^CD11c^+^. (**G**) Microglia were not changed by genotype or laser. (**H**–**J**): MHCII^−^, CD11c^−^, and CD11c^+^ macrophages were equally increased by laser in wildtype (blue) and *Il6*^−/−^ (red) mice (* = p < 0.05, ** = p < 0.01, *** = p < 0.001, N = 5–9 per group). (**K**) Dendritic cells were also increased by laser in both wildtype and *Il6*^−/−^ mice.

Because macrophages produce IL6 and classical monocyte-derived macrophages are necessary for CNV pathogenesis^11,12^, we examined macrophage numbers after laser injury. We subjected 10–12 week old female wildtype and *Il6*^−/−^ mice to laser injury. On Day 3, we harvested whole eyes (cornea, sclera, iris, ciliary body, lens, retina, choroid, and RPE), and performed multi-parameter flow cytometry to measure ocular macrophage numbers from unlasered and lasered mice. We analyzed whole eyes to reduce variance created by uneven dissections and maximize the rigor and reproducibility of our data. Our full gating strategy and fluorescence minus one (FMO) controls for this panel were previously published^13^. We identified ocular leukocytes using CD45^+^ staining (Fig. 4a). We captured mononuclear phagocytes and excluded B cells (B220), T cells (CD4, CD8), eosinophils (SiglecF), neutrophils (Ly6G), and NK cells (NK1.1) by CD11b^+^Lin^−^ staining (Fig. 4b). We used the quantitative amount of CD45 to differentiate CD45^dim^ microglia^11,13,14^ from CD45^high^ macrophages and dendritic cells (Fig. 4c). We found no change in microglia numbers (CD45^dim^CD64^+^MHCII^low^) between wildtype and *Il6*^−/−^ mice in both unlasered and lasered groups (Fig. 4d,g). By contrast, MHCII^−^ macrophages (CD45^high^CD64^+^MHCII^−^) were increased by laser 13-fold (p < 0.05) in wildtype and 15-fold (p < 0.01) in *Il6*^−/−^ mice with no significant difference between groups (Fig. 4e,h). Additionally, CD11c^−^ (CD45^high^CD64^+^MHCII^+^CD11c^−^) and CD11c^+^ (CD45^high^CD64^+^MHCII^+^CD11c^+^) macrophage numbers elevated with laser by five–eightfold with no significant changes between wildtype and *Il6*^−/−^ mice (Fig. 4f,i–j). Finally, dendritic cells (CD45^high^CD64^−^MHCII^+^CD11c^+^) increased fourfold (p < 0.001) in wildtype and sevenfold (p < 0.001) in *Il6*^−/−^ mice with no significant difference by genotype (Fig. 4f,k). These results demonstrate that although *Il6*^−/−^ mice have reduced CNV area, macrophage numbers and heterogeneity are unchanged compared to wildtype mice.

**Figure 5:**
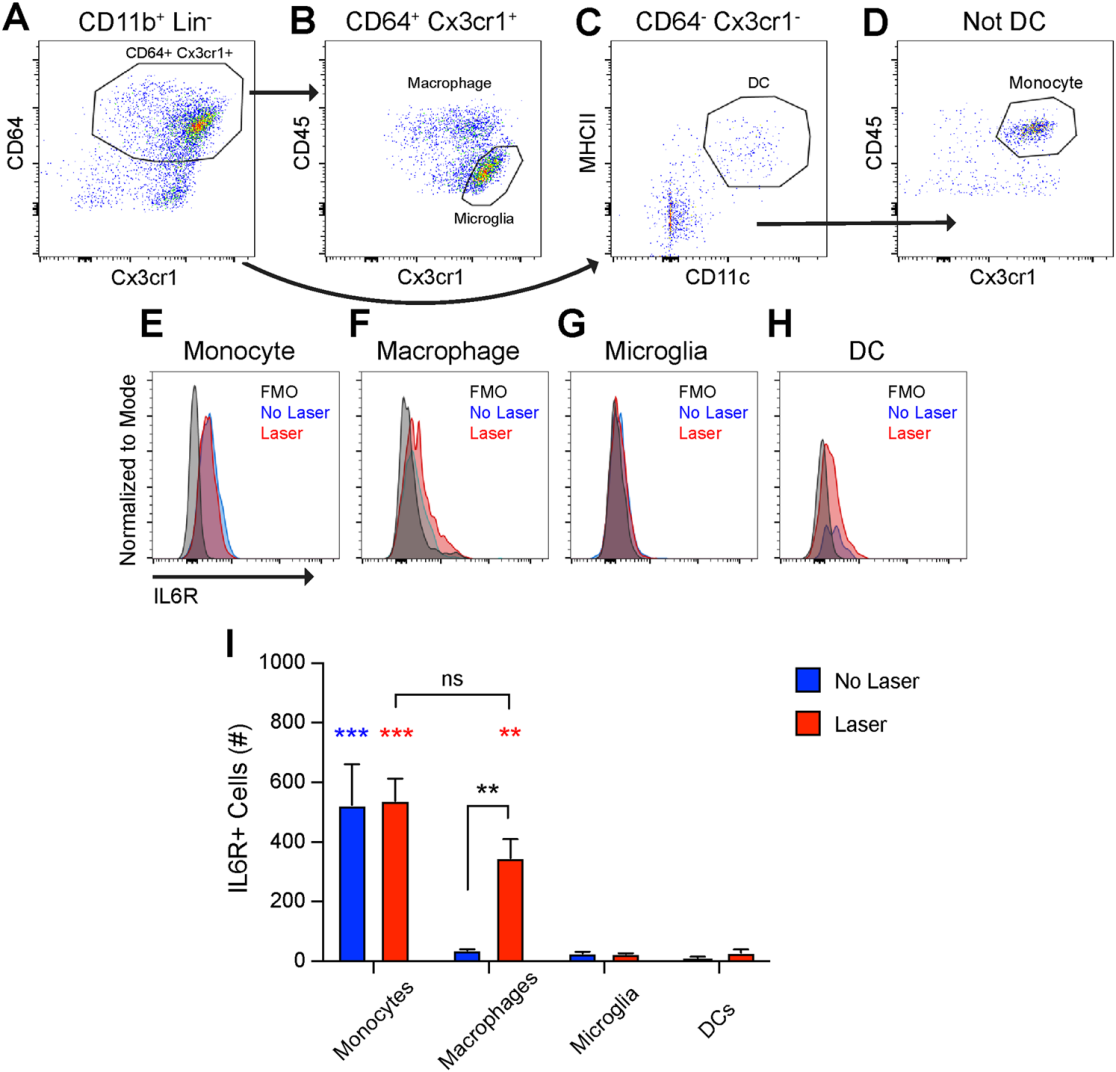
IL6R is expressed in ocular monocytes and macrophages. (**A**) CD64^+^Cx3cr1^+^ cells were gated forward. (**B**) Microglia were defined as CD45^dim^Cx3cr1^+^ while all other cells were determined to be macrophages (Boolean gate). (**C**) Non-Cx3cr1^+^CD64^+^ cells were gated forward from (A) and dendritic cells (DC) were defined as MHCII^+^CD11c^+^. (**D**) Non-MHCII^+^CD11c^+^ cells from (C) were gated forward and monocytes were defined as CD45^high^Cx3cr1^+^. E–H: Representative frequency histograms for IL6R expression from the fluorescence minus one (FMO) control, untreated (No Laser, Blue), and lasered (Laser, Red) mice in monocytes (**E**), macrophages (**F**), microglia (**G**), and dendritic cells (DC, **H**). I: At steady state, monocytes expressed the IL6R. The number of IL6R^+^ macrophages were increased by laser (** = p < 0.01, *** = p < 0.001, blue asterisks indicate differences from unlasered mice, red asterisks indicate differences from lasered mice, N = 3 per group).

IL6 classically binds to cell surface IL6 receptor (IL6R) leading to intracellular signaling transduction via the gp130 coreceptor^15^. The IL6R is known to be expressed in leukocytes^15^, but has not been investigated in the eye. We used multi-parameter flow cytometry to identify IL6R expression. Wildtype 10–12 week old female mice were subjected to laser treatment, and eyes were harvested on Day 3. Mononuclear phagocytes were identified as CD45^+^CD11b^+^Lin^−^ identically to Fig. 4A,B. CD64^+^Cx3cr1^+^ cells were gated forward (Fig. 5a), and microglia were defined as CD45^dim^Cx3cr1^high^ while all other CD64^+^Cx3cr1^+^ cells (Boolean gate) were delineated as ocular macrophages (Fig. 5b). Dendritic cells were identified from CD64^−^Cx3cr1^−^ cells as MHCII^+^CD11c^+^ (Fig. 5c). The non-DC population from Fig. 5c was gated forward (Boolean gate) and CD45^high^Cx3cr1^+^ cells were defined as monocytes (Fig. 5d). The major populations of ocular IL6R^+^ cells were monocytes and macrophages. At steady state, the number of IL6R^+^ monocytes were greater than all other groups (Fig. 5e,i, p < 0.001). After laser injury, IL6R^+^ monocytes were unaffected and were significantly more than all other groups except for macrophages (p < 0.001). The number of IL6R^+^ macrophages were increased 9.3-fold (p < 0.01) by laser treatment, and were significantly greater than microglia and dendritic cell counts (Fig. 5i, p < 0.01). Few IL6R^+^ microglia or dendritic cells were detected at steady state or after laser injury (Fig. 5g–i). These findings show that monocytes and macrophages are the major populations of ocular IL6R^+^ cells after laser injury.

**Figure 6:**
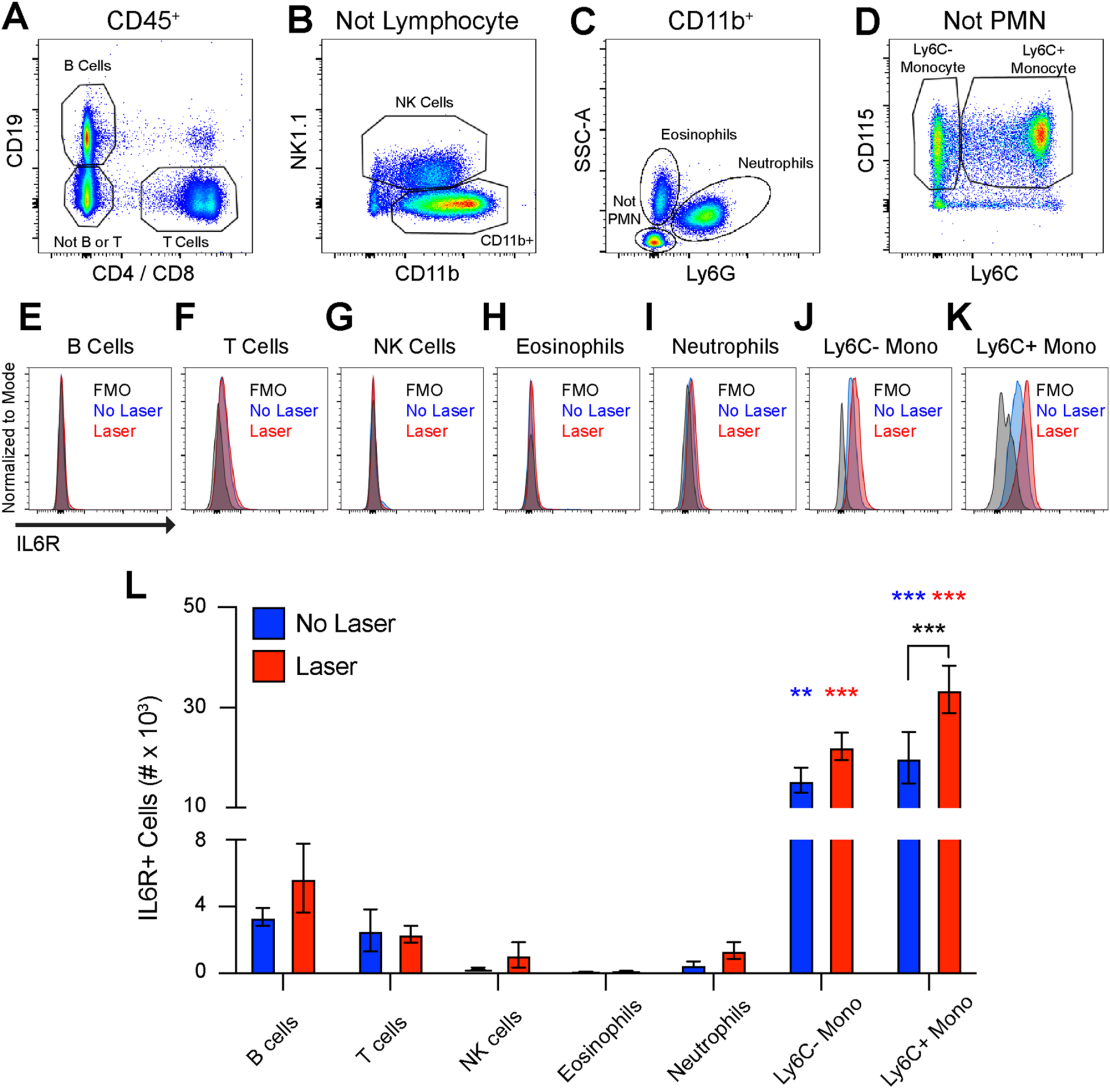
Monocytes express the IL6R in peripheral blood. (**A**) B cells were defined as CD19^+^, T cells were defined as CD4^+^ or CD8^+^, and non-lymphocytes (not B or T) were gated forward. (**B**) NK cells were delineated as NK1.1^+^ and CD11b^+^NK1.1^−^ cells were gated forward. (**C**) Neutrophils were identified as Ly6G^+^SSC^med^, eosinophils were found to be SSC^high^Ly6G^−^, and non-granulocytes (Not PMN) were gated forward. (**D**) Monocytes were defined as CD115^+^Ly6C^−^ or CD115^+^Ly6C^+^. (**E**–**K**) Representative frequency histograms for IL6R expression from the fluorescence minus one (FMO) control, untreated (No Laser, Blue), and lasered (Laser, Red) mice in B cells (**E**), T cells (**F**), NK cells (**G**), Eosinophils (**H**), Neutrophils (**I**), Ly6C^−^ monocytes (**J**), and Ly6C^+^ monocytes (**K**). L: All cells other than monocytes were < 5% IL6R^+^. At steady state, Ly6C^−^ and Ly6C^+^ monocytes expressed more IL6R than all other groups (** = p < 0.01, *** = p < 0.001 vs all other groups [Blue vs No Laser], N = 5–6 per group). After laser injury, more IL6R^+^Ly6C^−^ and IL6R^+^Ly6C^+^ monocytes detected compared to all other groups (*** = p < 0.001 vs all other groups [Red vs Laser], N = 5–6 per group). The number of IL6R^+^Ly6C^+^ monocytes were increased by laser treatment (*** = p < 0.001, Black: No Laser vs Laser, N = 5–6 per group).

In order to confirm our ocular monocyte findings, we performed multi-parameter flow cytometry on peripheral blood from unlasered and lasered mice. Wildtype 10–12 week old female mice were subjected to laser and peripheral blood was obtained on Day 3. B cells (CD19) and T cells (CD4, CD8) were identified from CD45^+^ cells (Fig. 6a). CD45^+^CD19^−^CD4^−^CD8^−^ cells were gated forward and NK cells were delineated as NK1.1^+^ (Fig. 6b). Neutrophils were characterized as Ly6G^+^SSC^med^ and eosinophils were defined as Ly6G^−^SSC^high^ from CD45^+^CD11b^+^NK1.1^−^ cells (Fig. 6c). Non-granulocytes were gated forward and identified as Ly6C^−^CD115^+^ or Ly6C^+^CD115^+^ monocytes (Fig. 6d). We found no change in the total number of B cells, T cells, NK cells, eosinophils, neutrophils, Ly6C^+^ monocytes, or Ly6C^−^ monocytes after laser injury (Fig S2). B cells, T cells, NK cells, eosinophils, and neutrophils were < 5% IL6R^+^ , found at low numbers, and were unchanged by laser (Fig. 6e–i, 6l). At steady state, significantly more IL6R^+^Ly6C^−^ (p < 0.01) and IL6R^+^Ly6C^+^ (p < 0.001) monocytes were detected compared to all other groups with no difference between Ly6C^−^ and Ly6C^+^ monocytes (Fig. 6j–l). After laser injury, IL6R^+^Ly6C^−^ monocytes were unchanged and remained significantly more than all other groups (p < 0.001, Fig. 6j,l). Alternatively, the number of IL6R^+^Ly6C^+^ monocytes increased with laser treatment (p < 0.001), and were significantly greater than all other groups (p < 0.001, Fig. 6k–l)). Since the total number of Ly6C^+^ monocytes was unchanged with laser, these data suggest that laser stimulated existing Ly6C^+^ blood monocytes to express the IL6R. These data confirm that monocytes are IL6R^+^ cells and constitute the overwhelming majority of IL6R^+^ blood cells in the context of a laser-injury model.

**Figure 7:**
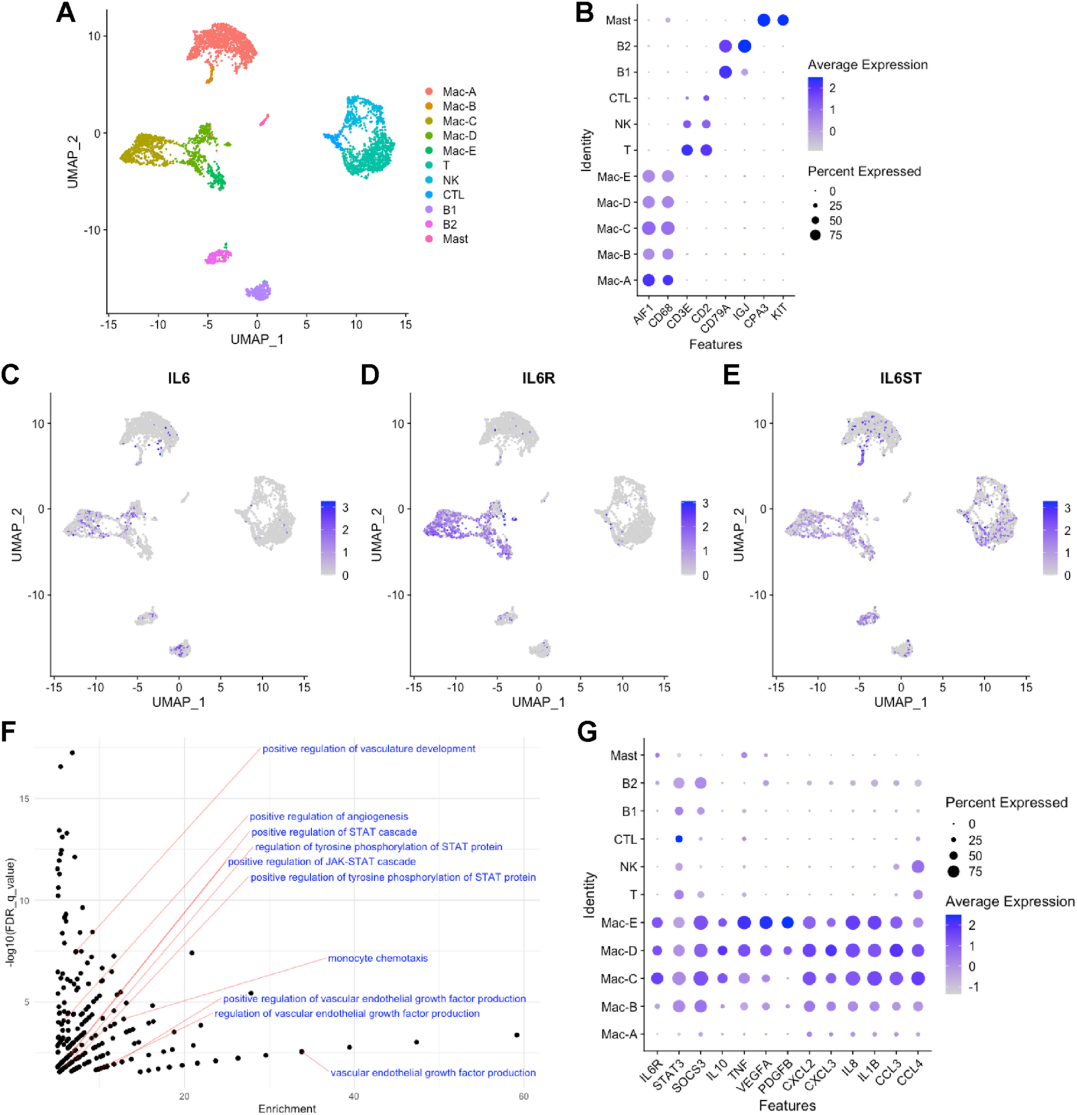
Single-cell RNA-seq of human choroid. (**A**) UMAP dimension plot of each cell from 7 human donors. (**B**) Dot Plot for identification of cell types. (**C**–**E**) Feature Plot of *IL6*, *IL6R*, and *IL6ST* expression. (**F**) Scatter plot (q-value vs enrichment) of GO terms with labels. (**G**) Dot Plot of genes that drove GO term enrichment for STAT signaling, angiogenesis, and monocyte chemotaxis.

In order to investigate IL6 and IL6R expressing cells in humans, we re-analyzed a recently published single-cell RNA-seq data set from human RPE-choroid samples^16^. We integrated the data from all 7 patients, performed cell clustering with Seurat v3^17,18^, and visualized the clusters using the uniform manifold approximation and projection (UMAP) technique (Fig. 7a). We identified 5 subsets of IBA1(*AIF1*)^+^*CD68*^+^ macrophages, 3 subtypes of *CD3E*^+^ or *CD2*^+^ T cells, 2 populations of *CD79A*^+^*IGJ*^+^ B cells, and *CPA3*^+^*KIT*^+^ mast cells (Fig. 7a,b). *IL6* expression was primarily detected in macrophages, and a few B cells (Fig. 7c). The *IL6R* was expressed predominantly by macrophage subsets Mac-C, Mac-D, and Mac-E (Fig. 7d). The IL6 coreceptor gp130 (*IL6ST*) demonstrated expression in all cell types (Fig. 7e).

We performed differential expression analysis between *IL6R*^+^ Mac-C, Mac-D, and Mac-E subtypes and all other human choroidal immune cells. We identified 244 genes up-regulated > twofold and 46 genes down-regulated < 0.5 fold (Table S1). Gene ontology (GO) enrichment analysis of up-regulated genes displayed enrichment for 4 GO terms associated with STAT signaling, 5 pro-angiogenic GO terms, and monocyte chemotaxis (Table S2, Fig. 7f). STAT3 is the principal signal transducer downstream of the IL6R^19^, and was expressed in Mac-C, Mac-D, and Mac-E (Fig. 7g). STAT3 target genes^20^ *SOCS3* (suppressor of cytokine signaling 3, 2.3-fold), *IL10* (interleukin-10, 3.1-fold), *TNF* (tumor necrosis factor, 2.9-fold), and *VEGFA* (3.0-fold) were up-regulated in Mac-C, Mac-D, and Mac-E compared to all other cells (Fig. 7g). In addition to being STAT3 target genes, IL10^21^, TNF^22^, and VEGFA^1,23^ are pro-angiogenic during CNV. Furthermore, *PDGFB* (platelet derived growth factor B, 2.1-fold), *IL1B* (interleukin-1 beta, sixfold), and the ELR chemokines CXCL2 (3.5-fold), CXCL3 (3.2-fold), and IL8/CXCL8 (5.0-fold), which all stimulate angiogenesis^24,25^, displayed increased expression in Mac-C, Mac-D, and Mac-E (Fig. 7g). Finally, *IL6R*^+^ macrophages demonstrated increased expression of the chemokines *CCL3* (3.5-fold) and *CCL4* (2.1-fold), which can attract pro-angiogenic monocytes^11,12^. These data suggest that human macrophages express IL6, the IL6R, and the IL6 coreceptor (gp130/*IL6ST*). Furthermore, *IL6R*^+^ human macrophages demonstrate a transcriptional profile consistent with STAT3 activation and angiogenesis.

## Discussion

**Figure 8:**
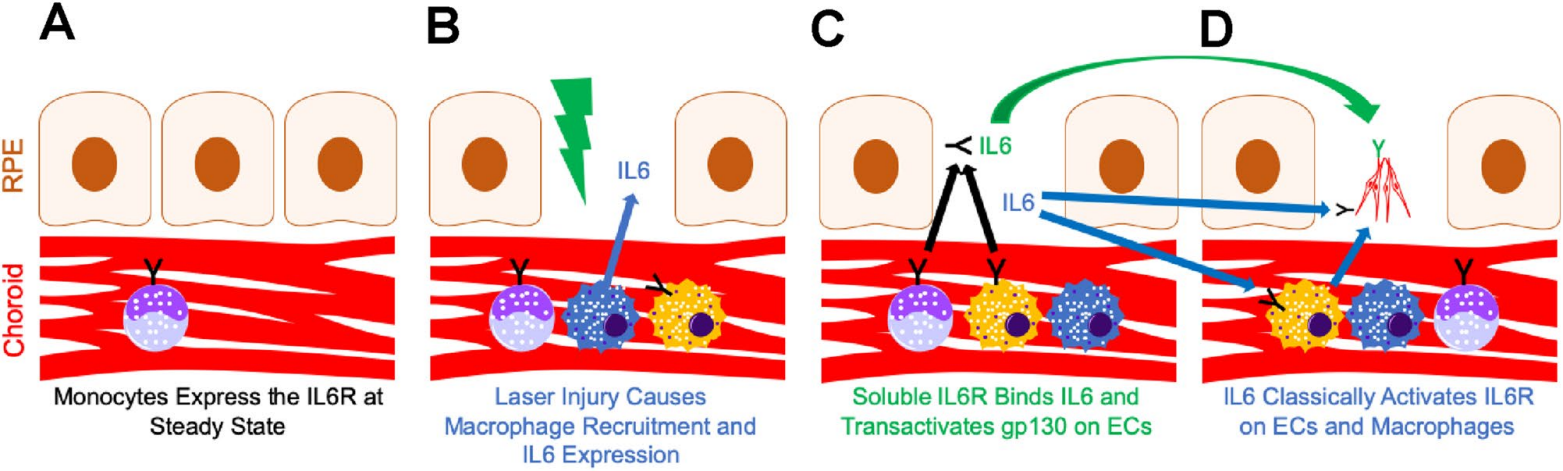
Model of IL6 signaling during CNV. (**A**) At steady state, IL6 is not expressed and the IL6R (Black Y) is expressed by monocytes (purple). (**B**) Laser injury (lightning bolt) causes macrophage influx into the choroid including IL6-expressing macrophages (blue) and IL6R^+^ macrophages (yellow with black Y). (**C**, **D**) Soluble IL6R derived from monocytes and macrophages binds macrophage-derived IL6 and trans-activates (green arrow) gp130 (Green Y) on CNV endothelial cells. Macrophage-derived IL6 classically activates (blue arrow) the IL6R (Black Y) on endothelial cells and macrophages. D: IL6R^+^ macrophages stimulate angiogenesis (blue arrow).

In this report, we used the choroid sprouting and laser-induced CNV models to investigate the IL6 pathway during choroidal angiogenesis. At steady state, no IL6 was detectable in the choroid, while monocytes expressed the IL6R (Fig. 8a). After laser injury, macrophages are recruited to the laser injury site, produce IL6, and express the IL6R (Fig. 8b). IL6 expression is both necessary and sufficient for choroidal angiogenesis. The likely mechanism by which IL6 stimulates angiogenesis is by classical activation of IL6R^+^ macrophages to indirectly stimulate angiogenesis (Fig. 8d).

Our data demonstrate that macrophages produce IL6 in the laser-induced CNV model (Fig. 1). Because IL6 is a secreted peptide, we used RNAscope to identify intracellular *Il6* mRNA in order to delineate the cellular source of IL6. Our findings are in agreement with prior immunofluorescent staining of IL6 protein using CD11b^26^ and *Cx3cr1*-GFP^27^ co-labeling in the choroid. In addition, murine bone marrow-derived monocytes^28^ and human alveolar macrophages^29^ express and secrete IL6 protein. Therefore, our data add a new methodology to the literature supporting macrophages as a key source of IL6 production in the eye.

Since IL6 is pro-inflammatory and pro-angiogenic cytokine^10^, we investigated the function of IL6 during choroidal angiogenesis. A prior group showed that male *Il6*^−/−^ mice display a 30% reduction in laser-induced CNV area^19^. Similarly, we demonstrate that *Il6*^−/−^ mice have a 42% reduction in CNV area in both male and female mice, with no sex-specific effects (Figs. 2 & S1). Additionally, we find that IL6 is sufficient to stimulate angiogenesis in the choroidal sprouting assay (Fig. 3). These data are similar to prior results that IL6 can stimulate angiogenesis in the aortic ring assay^10^, and promote endothelial cell line motility in the scratch wound assay^30^. Furthermore, human studies show that intraocular IL6 levels are associated with nAMD activity^31^, and systemic IL6 levels correlate with progression to advanced AMD^32^. Thus, our data show that IL6 is necessary and sufficient for choroidal angiogenesis in mice, and human studies support a pathogenic role for IL6 in nAMD.

The mechanism by which IL6 stimulates angiogenesis is unclear. IL6 uses both classical and trans-activation to exert its signaling effects^15^. In the classical pathway, IL6 binds to cell surface IL6R and signals intracellularly via gp130. Our data support a mechanism where macrophage-derived IL6 classically activates IL6R^+^ macrophages, leading to macrophage-driven angiogenesis (Fig. 8d). IL6 was detected in mouse (Fig. 1) and human (Fig. 7) macrophages. After laser injury in mice, IL6R^+^Ly6C^+^ monocytes are increased (Fig. 6), and IL6R^+^ macrophages are recruited to the eye (Fig. 5). In support of this mechanism, multiple studies have shown that macrophages, and specifically Ly6C^+^ classical monocyte-derived macrophages, stimulate angiogenesis during laser-induced CNV^11,12,33^. Additionally, human choroidal macrophages expressed both *IL6R* and *IL6ST* (Fig. 7), demonstrating their ability to respond to the IL6 stimulus. Finally, *IL6R*^+^ human macrophages demonstrated a transcriptional profile consistent with STAT3 activation, angiogenesis, and monocyte chemotaxis (Fig. 7f–g). These data support a mechanism where macrophage-derived IL6 stimulates IL6R^+^ macrophages to drive angiogenesis.

Our data do not exclude an additional mechanism where macrophage-derived IL6 classically signals to endothelial cells to promote angiogenesis (Fig. 8d). We did not investigate IL6R or co-receptor expression in endothelial cells. However, prior reports have shown IL6R expression on primary and cultured endothelial cells^10^. Furthermore, IL6 addition to aortic ring assays^10^ or the choroidal sprouting assay (Fig. 3) stimulates angiogenesis. This effect could be directly on endothelial cells, but both aortic and choroidal tissue contain macrophages. These data suggest that macrophage-derived IL6 could signal classically through IL6R^+^ endothelial cells to increase angiogenesis.

Trans-signaling is a third potential mechanism of IL6-driven angiogenesis (Fig. 8c). Our data show that monocytes are the major IL6R^+^ cells in the eye and blood (Fig. 5–6). In human choroid, we did not detect monocytes, but macrophages were also the major IL6R^+^ cell type (Fig. 7). IL6 may bind monocyte/macrophage-derived soluble IL6R and stimulate angiogenesis via trans-activation of endothelial cell gp130/*IL6ST*. In support of this model, exogenous IL6 and soluble IL6R stimulate angiogenesis via trans-signaling and downstream activation of STAT3 and Ca^2+^/calmodulin-dependent protein kinase II$\updelta ($CaMKII$\updelta$) in endothelial cells^30^. Furthermore, conditional STAT3 and CaMKII$\updelta$ endothelial cell knockout mice demonstrate decreased retinal vascular development^30^. These data support a third possible mechanism of IL6-driven angiogenesis. Future studies using conditional IL6R knockout mice will be necessary to further elucidate the mechanism of IL6-driven angiogenesis.

In summary, we demonstrate that IL6 is up-regulated by laser injury and expressed by macrophages at the laser injury site. Additionally, IL6 is necessary and sufficient for choroidal angiogenesis. These findings support IL6 and the IL6R as possible therapeutic targets in nAMD. Finally, IL6 stimulates choroidal angiogenesis through one of three possible mechanisms: IL6 classical activation of macrophages to indirectly stimulate angiogenesis, IL6 binding to monocyte-derived soluble IL6R to trans-activate endothelial cells, and IL6 classical signaling of endothelial cells to directly stimulate angiogenesis.

## Methods

### Animals

Breeding pairs of wildtype (C57BL6/J; #000664), and *Il6*^*−/−*^ (B6.129S2-*Il6*^*tm1Kopf*^/J; 002650) were obtained from Jackson Labs (Bar Harbor, ME). Wildtype and *Il6*^*−/−*^ animals used in this study were first- or second-generation crosses of parental mice. One complete litter from each breeding pair was genotyped to confirm the correct genotype and the absence of the RD8 allele (*Crb1*). Genotyping services were performed by Transnetyx (Cordova, TN). All experiments were performed on 10–12 week old mice. Mice were housed in a specific pathogen free, barrier facility and maintained on a 12 h light/dark cycle. All experiments were conducted in accordance with the ARRIVE guidelines and were approved by the Northwestern University Institutional Animal Care and Use Committee.

### Laser-induced CNV

Male and female 10–12 week-old mice were treated as previously described^6,11^. Briefly, mice were anesthetized with a ketamine/xylazine (Akorn, Lake Forest, IL) cocktail. Pain control and hydration were achieved with a 1 mg/kg subcutaneous injection of Meloxicam (Henry Schein Animal Health, Melville, NY). Eyes were anesthetized, dilated, and a cover slip was coupled to the cornea with Gonak (Akorn) for slit lamp microscopy and laser. Four (immunofluorescence) or eight (flow cytometry, immunohistochemistry and ELISA; to increase inflammatory cell numbers) focal burns (75 μm, 110 mW, 100 ms) were administered in each eye using a 532 nm argon ophthalmic laser (IRIDEX, Mountain View, CA) via a slit lamp delivery system (Zeiss, Oberkochen, Germany).

### RNA scope

Enucleated eyes from control and Day 3 after laser were placed in modified Davidson’s buffer (11% v/v Glacial acetic acid, 2.2% v/v neutral buffered formalin, 32% v/v ethanol [all Millipore-Sigma, St. Louis, MO] at least 1 ml / eye) overnight at room temperature. Samples were transferred to 10% neutral buffered formalin for 2 h at room temperature, then stored in 70% ethanol until embedding in paraffin. Tissue blocks were cut at 4 μm thickness. RNAscope using a probe against mouse *Il6* (#315,898, ACDBio, Newark, CA) was performed using manufacturer’s protocol (#322,360, ACDBio). Negative controls included no probe on wildtype slides, and the *Il6* probe on *Il6*^−/−^ eyes. All slides were counter stained with hematoxylin.

### Immunohistochemistry

Eyes were paraffin embedded identically to RNAscope. Slides were deparaffinized on an automated platform (Leica Autostainer XL, Leica Microsystems, Buffalo Grove, IL), then processed for antigen retrieval (Sodium Citrate pH 6 for 10 min at 110 °C). Slides were incubated with primary antibodies shown in Table 1 (Iba1, 1:1000; F4/80, 1:500) in PBS overnight at 4 °C in a humidified chamber. After washing with PBS, slides were incubated with the appropriate secondary antibody via an automated system (Biocare Intellipath, Pacheco, CA). Red chromogen development was completed with a Warp Red Chromogen kit (Biocare). Positive (brain and spleen) and no primary controls were used to confirm activity. All slides were counter stained with hematoxylin.

**Table 1:** Antibodies used in this study.

Antibody	Fluorophore	Clone	Usage	Manufacturer
Rat anti-mouse CD16/CD32	N/A	2.4G2	F_c_ block	BD Biosciences
Mouse anti-mouse CD64	PE	X54-5/7.1	Eye	BioLegend
Hamster anti-mouse CD11c	BV 421	HL3	Eye^a^	BD Biosciences
Rat anti-mouse Ly6G	PE-CF594	1A8	Eye	BD Biosciences
Mouse anti-mouse NK1.1	PE-CF594	PK136	Eye	BD Biosciences
Rat anti-mouse Siglec F	PE-CF594	E50-2440	Eye^a^	BD Biosciences
Rat anti-mouse B220	PE-CF594	RA3-6B2	Eye	BD Biosciences
Rat anti-mouse CD8	PE-CF594	53–6.7	Eye and blood	BD Biosciences
Rat anti-mouse CD4	PE-CF594	RM4-5	Eye and blood	BD Biosciences
Rat anti-mouse MHC II	AlexaFluor 700	M5/114.15.2	Eye	BioLegend
Rat anti-mouse CD11b	APC-Cy7	M1/70	Eye^a^	BD Biosciences
Rat anti-mouse CD45	PE-Cy7	30-F11	Eye^a^	BD Biosciences
Rat anti-mouse CD45	FITC	30-F11	Eye and blood^a^	BD Biosciences
Rat anti-mouse IL6R	PE-Cy7	AL-21	Eye and blood^a^	BD Biosciences
Rat anti-mouse CD31	BB700	MEC 13.3	Eye^a^	BD Biosciences
Mouse anti-mouse Cx3cr1	AlexaFluor 647	SA011F11	Eye	BioLegend
Rat anti-mouse Ly6G	PerCP-Cy5.5	1A8	Blood^a^	BD Biosciences
Rat anti-mouse CD11b	eFluor 450	M1/70	Blood^a^	Invitrogen
Rat anti-mouse CD19	APC	1D3	Blood^a^	BD Biosciences
Mouse anti-mouse NK1.1	AlexaFluor 700	PK136	Blood	BD Biosciences
Rat anti-mouse CD115	PE	AFS98	Blood	Invitrogen
Rat anti-mouse Ly6C	APC-Cy7	AL-21	Blood^a^	BD Biosciences
Rat anti-mouse CD19	PE	1D3	Compensation	BD Biosciences
Rat anti-mouse CD19	AlexaFluor 700	1D3	Compensation	BD Biosciences
Fixable viability dye	eFluor 506	N/A	Eye^a^	Invitrogen
Rat anti-mouse CD102 (ICAM2)	N/A	3C4(mIC2/4)	Immunofluorescence	BD Biosciences
Donkey anti-rat (H + L)	AlexaFluor 488	N/A	Immunofluorescence	Invitrogen
Rabbit anti-mouse Iba1	N/A	EPR16588	Immunohistochemistry	Abcam
Rabbit anti-mouse F4/80	N/A	D2S9R	Immunohistochemistry	Cell Signaling
Donkey anti-rabbit (H + L)	Alkaline Phosphatase	N/A	Immunohistochemistry	Abcam

^a^Antibody used for compensation setup.

### ELISA

Enucleated eyes from control and Day 3 post laser treatment were placed in cold PBS with protease inhibitor (ThermoFisher Scientific, #87,786, Waltham, MA) before dissection of optic nerve, extraocular muscles, orbital tissue and conjunctiva. A circumferential incision was made at the limbus to remove cornea, iris, ciliary body and lens. The resulting posterior eye cups containing retina, choroid, vitreous, and sclera were placed in a dry dissecting dish and cut into 4–8 pieces before transfer of solids and residual liquid into a new 1.7 ml microcentrifuge tube. Samples were spun at 1000 × g for 30 s at 4 °C before removal of any clear supernatant. Pelleted samples were resuspended in 100 μl RIPA buffer (Cell Signaling, Danvers, MA) + protease inhibitor for mechanical disruption via disposable plastic mortar (Kimble #749,521–1500, Millville, NJ) and motorized grinding (Fisher Scientific, #12–141-361, Pittsburgh, PA). Homogenized samples were placed on ice for 15 min then centrifuged for 3 min at 14,000 × g at 4 °C. Supernatants were harvested for analysis via endpoint DuoSet ELISA for IL6 (DY406, R&D Systems, Minneapolis, MN) using manufacturer’s instructions. Plates were read (Bio-Rad, Hercules, CA) and standard curves and quantification of samples were conducted using the interpolation function of a 4 parameter logistic curve fit in Prism (Graphpad Software LLC).

### Choroidal sprouting assay

Assays were performed as previously described^6^. Briefly, enucleated eyes from 10–12 week old wildtype male mice were dissected until only a posterior eyecup of sclera, choroid and retinal pigmented epithelium (RPE) remained. Only the peripheral choroid was used for assays. 0.5–1.0 mm choroidal specimens were plated in Growth Factor Reduced Matrigel (#356,231, Corning, Bedford, MA). Depleted (2.5% FBS and 1:200 supplements) EGM2-MV medium (CC-3162, Lonza, Walkersville, MD) was changed every 2 days. Exogenous IL6 (R&D Systems, Minneapolis, MN) in growth media was first added on Day 2. Media with and without IL6 was changed every two days and pictures were taken on Days 4–7. Growth area was measured via Nikon software as previously described^6^.

### Immunofluorescence

Eyes were treated as previously described^6,11^. Briefly, mice were sacrificed 2 weeks after laser-induced CNV. Enucleated eyes were fixed for 1 h in 1% paraformaldehyde (#15,713-S, Electron Microscopy Sciences, Hatfield, PA) at room temperature. Eyes were washed in Tris-buffered saline (TBS) and dissected to remove conjunctiva, cornea, iris, ciliary body, lens, and retina leaving a posterior eye cup of RPE, choroid, and sclera. Eye cups were blocked in TBS + 5% Donkey serum (S30, Sigma-Aldrich), then treated with an anti-ICAM-2 primary antibody (1:500, Table 1), and Alexa Fluor 488-conjugated anti-rat secondary antibody (Table 1). Pictures were captured on a Ti2 widefield microscope (Nikon, Melville, NY). Area was quantified using Fiji by a reviewer blinded to animal identification.

### Flow cytometry of whole eyes

Experiments were performed as previously described^13^. Briefly, female mice (unlasered control and Day 3 post laser) were sacrificed, and enucleated eyes were placed into HBSS. Animals were not perfused as we previously demonstrated no change in macrophage numbers at steady state or after laser injury with or without systemic perfusion^13^. Eyes were cleaned of optic nerve, extraocular muscles, orbital tissue, and conjunctiva. Whole mouse eyes including cornea, sclera, iris, ciliary body, lens, vitreous, retina, RPE, and choroid, were minced into small pieces. Eye pieces were further mechanically and chemically digested before passing through a 40 μm filter to obtain a single cell suspension. Cell suspensions were stained for live cells and washed. Cell suspensions were blocked and stained with cell surface antibodies found in Table 1. Both eyes were pooled from one mouse to determine cells per mouse, using counts beads. Samples were run on a modified LSRII (BD Biosciences, San Jose, CA) and analyzed using FlowJo v10. Please see our prior publication for all fluorescence minus one controls^13^.

### Flow cytometry of peripheral blood

Experiments were performed as previously described^11^ on unlasered control and Day 3 post laser. Briefly, blood from freshly sacrificed animals was obtained via cardiac puncture. Samples were placed in EDTA tubes (Sarstedt, Numbrecht, Germany) to prevent clotting. Cells were blocked and stained by an antibody cocktail (Table 1). Stained cells were fixed and red blood cells were lysed using FACSLyse (BD Biosciences). After washing, samples were resuspended in MACS buffer (Miltenyi Biotec, Auburn, CA) and run on a modified LSRII with subsequent analysis using FlowJo v10.

### Bioinformatics

Gene expression data (.tsv files) from human choroidal samples were downloaded from the GEO database (GSE135922). Data was imported into Seurat v3^17,18^. The FindIntegrationAnchors followed by the IntegrateData functions (dims 1:50) were used to integrate the data into one data set and perform batch corrections. The data were rescaled (ScaleData function), and principal component analysis (PCA) was performed (RunPCA, npcs = 50). The Elbow Plot technique was used to identify 19 significant principal components (PCs). Cells were clustered using FindNeighbors (dims = 1:19) followed by FindClusters (resolution = 0.4). The RunUMAP function was used to visualize the cell clusters. Differential expression and cell identification were performed using FindAllMarkers (min.pct = 0.25, logfc.threshold = log2). The FeaturePlot and DotPlot functions were used to visualize gene expression. FindMarkers (min.pct = 0.25, logfc.threshold = log2) was used to generate a list of differentially expressed genes between Mac-C, Mac-D, Mac-E and all other immune cells. Gene ontology (GO) enrichment analysis was performed on up-regulated genes independently using a fold change cut-off = > 2.0, and adjusted p-value < 0.001. GOrilla was used for GO enrichment^34,35^, using a background of genes expressed only in choroidal leukocytes. GO terms were visualized using ggplot that met a number of genes (b) > 2, enrichment > 5, and false discovery rate (FDR) q-value < 0.05.

### Statistical Analysis

Statistical analysis was performed in Prism. IL6 levels and CNV area in *Il6* null mice (PBS vs Propranolol) were compared by Student’s unpaired t-test. CNV area in wildtype vs in *Il6* null mice were compared by Welch’s t-test due to unequal variances between groups. Choroidal sprouting area analysis was performed using Two-Way ANOVA followed by Dunnett’s multiple comparisons test. Flow cytometry comparisons of macrophage numbers were made using the Brown-Forsythe and Welch ANOVA followed by Dunnett’s T3 multiple comparisons test due to unequal variances between unlasered and lasered mice. IL6R expression in eyes and blood was compared using Two-Way ANOVA followed by Tukey’s multiple comparisons test.

## Supplementary Information

Supplementary Information 1.

Supplementary Information 2.

Supplementary Information 3.

Supplementary Information 4.

Supplementary Information 5.

## Data Availability

The datasets used and analyzed for mouse studies are available from the corresponding author on reasonable request. All human data analyzed during this study are included in this published article and its supplementary information files.
