# Supplementary material for: Macrophage-derived interleukin-6 is necessary and sufficient for choroidal angiogenesis
Source: Sci Rep. 2021 Sep 10;11:18084. doi: 10.1038/s41598-021-97522-x (PMC8433398; doi:10.1038/s41598-021-97522-x)
Supplement: Supplementary file 1 — Supplementary Information 1. [file 41598_2021_97522_MOESM1_ESM.docx]

**Fig S1. IL6 was necessary for CNV in male and female mice**. (A) IL6-deficiency resulted in a 43% decrease in CNV area (p=0.06, N = 13-14 per group) in female mice. (B) IL6-deficiency resulted in a 38% decrease in CNV area (p=0.07, N = 10-12 per group) in male mice.

**Fig S2. Laser treatment did not affect peripheral blood cell populations.** The total number of B cells, T cells, NK cells, eosinophils, neutrophils, Ly6C-, and Ly6C+ monocytes was unaffected by laser injury (N=5-6 per group).

**Table S1. List of differentially expressed genes.** Excel file of gene names, cluster, adjusted p-value (p_val_adj), percent of cells expressing gene (pct.1 = current cluster, pct.2 = all other clusters), fold change (avg_logFC = natural log fold change), and raw p-value (p_val).

**Table S2.** **Table of GO terms**. Excel file of GO terms, description of GO term, p-value, FDR q-value, fold enrichment, number of genes expressed in macrophages (N), number of genes in GO term (B), number of differentially expressed genes (n), number of differentially expressed genes in the GO term (b), and the specific genes that were differentially expressed in the GO term.
