# Supplementary figures and images for: Macrophage-derived interleukin-6 is necessary and sufficient for choroidal angiogenesis
Source: Sci Rep. 2021 Sep 10;11:18084. doi: 10.1038/s41598-021-97522-x (PMC8433398; doi:10.1038/s41598-021-97522-x)

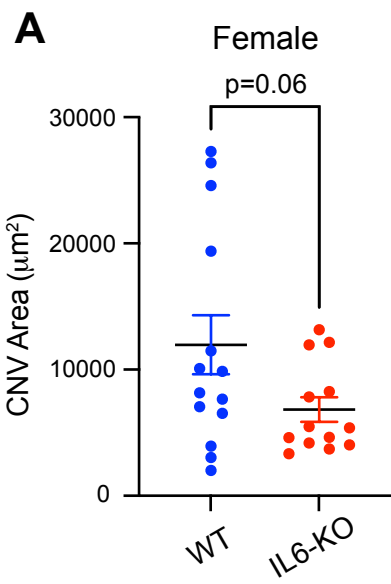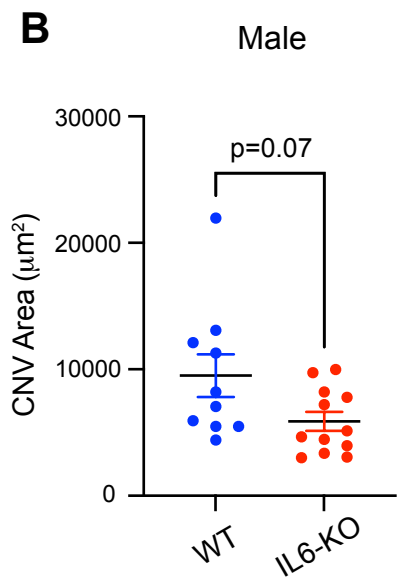

Supplement: Supplementary file 2 — Supplementary Information 2. [file 41598_2021_97522_MOESM2_ESM.pdf]

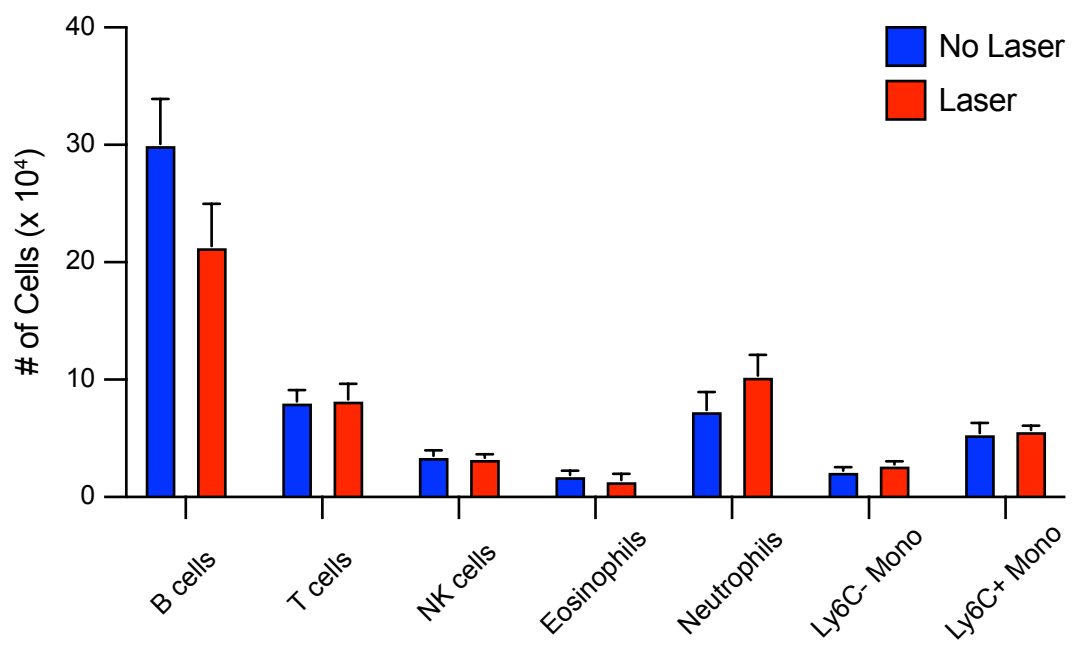

Supplement: Supplementary file 3 — Supplementary Information 3. [file 41598_2021_97522_MOESM3_ESM.pdf]
